# *catena*-Poly[{μ-η^5^:η^5^-1-[2-(dimethyl­amino)­ethyl-κ*N*]cyclo­penta­dien­yl}-lithium(I)-(μ-1,1,3,3-tetra-*tert*-butyl­triphosphane-κ^3^ *P* ^2^:*P* ^1^,*P* ^3^)lithium(I)]

**DOI:** 10.1107/S1600536810039759

**Published:** 2010-10-13

**Authors:** Rafal Grubba, Jaroslaw Chojnacki, Jerzy Pikies

**Affiliations:** a Chemical Faculty, Gdansk University of Technology, G. Narutowicza 11/12, Gdansk PL-80233, Poland

## Abstract

The title compound, [Li_2_(C_9_H_14_N)(C_16_H_36_P_3_)]_*n*_, is a by-product of the reaction of [Cp(C_5_H_4_CH_2_CH_2_NMe_2_)ZrCl_2_]_*n*_ with ^*t*^Bu_2_P–P(SiMe_3_)Li in toluene. It is a coordination polymer composed of infinite chains running along [010]. One Li(I) atom is chelated by the cyclo­penta­dienyl ring and and the N atom of the scorpionate ligand and a P atom, whereas the other Li(I) atom is coordinated by the backside of the cyclo­penta­dienyl ring and two P atoms. Both Li(I) atoms adopt a distorted trigonal coordination. The structure was determined from a twinned crystal, but only the data from the main twin component was used. The fraction of components in the crystal was 0.555:0.445 and the twin matrix corresponds to twofold rotation about the *c* axis (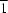
00/0
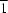
0/001).

## Related literature

For the synthesis, see: Chojnacki *et al.* (2007 ▶); Kovacs *et al.* (1996*b* ▶). For related structures, see: Kovacs *et al.* (1996*a* ▶); Kunz *et al.* (2000 ▶).
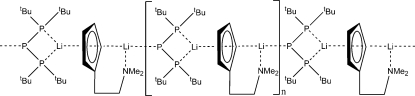


## Experimental

### 

#### Crystal data

[Li_2_(C_9_H_14_N)(C_16_H_36_P_3_)]
*M* *_r_* = 471.45
Monoclinic, 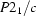

*a* = 8.9063 (6) Å
*b* = 18.8522 (8) Å
*c* = 19.3934 (17) Å
β = 115.314 (6)°
*V* = 2943.5 (3) Å^3^
*Z* = 4
Mo *K*α radiation
μ = 0.21 mm^−1^
*T* = 120 K
0.44 × 0.23 × 0.20 mm

#### Data collection

Oxford Diffraction Xcalibur diffractometer with a Sapphire2 detector
Absorption correction: multi-scan (*CrysAlis PRO*; Oxford Diffraction, 2009 ▶) *T*_min_ = 0.734, *T*_max_ = 1
14505 measured reflections
4818 independent reflections
3492 reflections with *I* > 2σ(*I*)
*R*_int_ = 0.093

#### Refinement

*R*[*F* ^2^ > 2σ(*F* ^2^)] = 0.095
*wR*(*F* ^2^) = 0.258
*S* = 1.13
4818 reflections
294 parameters
H-atom parameters constrained
Δρ_max_ = 0.72 e Å^−3^
Δρ_min_ = −0.61 e Å^−3^

### 

Data collection: *CrysAlis PRO* (Oxford Diffraction, 2009 ▶); cell refinement: *CrysAlis PRO*; data reduction: *CrysAlis PRO*; program(s) used to solve structure: *SHELXS97* (Sheldrick, 2008 ▶); program(s) used to refine structure: *SHELXL97* (Sheldrick, 2008 ▶); molecular graphics: *ORTEP-3* (Farrugia, 1997 ▶); software used to prepare material for publication: *WinGX* (Farrugia, 1999 ▶).

## Supplementary Material

Crystal structure: contains datablocks global, I. DOI: 10.1107/S1600536810039759/bt5361sup1.cif

Structure factors: contains datablocks I. DOI: 10.1107/S1600536810039759/bt5361Isup2.hkl

Additional supplementary materials:  crystallographic information; 3D view; checkCIF report

## Figures and Tables

**Fig. 1.**
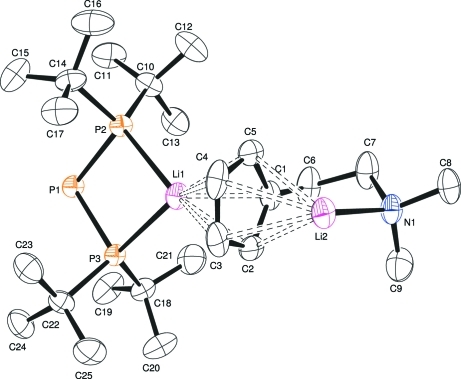
The asymmetric unit of the title compound. Ellipsoids are drawn at the 50% probability level.

**Fig. 2.**
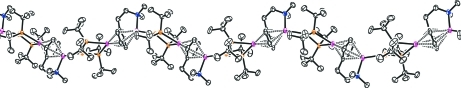
Coordination polymer of the title compound.

## supplementary crystallographic information

### Comment

The schematic structure of the linear coordination polymer of (I) is shown in Fig.1. The monomeric unit of the title compound consists of two lithium atoms and two ligands: (*^t^*Bu_2_P)_2_P and C_5_H_4_CH_2_CH_2_NMe_2_. Both lithium atoms adopt distorted trigonal geometry. Li1 atom is bonded in a η^5^-fashion from the one face of C_5_H_4_ ring system and is also bonded with two terminal phosphorous atoms P2, P3 from a (*^t^*Bu_2_P)_2_P ligand. Atom Li2 is coordinated by atom P1 from another triphosphane ligand and is η^5^-coordinated to the other face of the C_5_H_4_ ring system, assisted by κ*N*-coordination of the attached dimethylamino substituent. Atoms Li1, P1, P2, P3 form a planar ring (maximum deviation 0.069 Å). The two lithium atoms and C_5_H_4_ ring form an "inverse sandwich". The structure of (*^t^*Bu_2_P)_2_P ligand in complex (I) is similar to the one found for [Li(THF)_2_{η^2^-(*^t^*Bu_2_P)_2_P}] (Kovacs *et al.*, 1996*a*) with comparable P–P and P–Li distances. To the best of our knowledge only one example of lithium complex with substituted Cp ligand which exhibits κ*N* and η^5^-coordination to the same lithium atom is known (Kunz *et al.*, 2000). Li–N distance in complex [{C_5_H_4_CH(Ph)NMe_2_}Li]_n_ (Kunz *et al.*, 2000) and in the title compound are similar [2.227 (5) Å; 2.237 (12) Å].

### Experimental

The work was carried out using the standard vacuum-nitrogen line and Schlenk techniques. [Cp(C_5_H_4_CH_2_CH_2_NMe_2_)ZrCl_2_]_n_ and *^t^*Bu_2_P—P(SiMe_3_)Li ^.^ 3 THF were prepared according to the procedure described in the literature (Chojnacki *et al.*, 2007; Kovacs *et al.*, 1996*b*). Suspension of 0.105 g (0.289 mmole) [Cp(C_5_H_4_CH_2_CH_2_NMe_2_)ZrCl_2_]_n_ in 2 ml toluene was added dropwise into solution of 0.264 g (0.559 mmole) *^t^*Bu_2_P—P(SiMe_3_)Li.3THF in 2 ml of toluene at 233 K. The mixture immediately turned red. Then the volume was reduced to about 2 ml and the concentrate was stored for a few weeks at room temperature. After this time the solution yielded colourless crystals of (I).

### Refinement

The structure turned out to be a rotational twin, only the data from the main twin component was used. The fraction of components in crystal was 0.555 / 0.444. The twin matrix corresponds to 2-fold rotation about *c* axis (-1 0 0 / 0 -1 0 / 0 0 1).

### Crystal data

**Table:**

[Li_2_(C_9_H_14_N)(C_16_H_36_P_3_)]	*F*(000) = 1032
*M**_r_* = 471.45	*D*_x_ = 1.064 Mg m^−^^3^
Monoclinic, *P*2_1_/*c*	Mo *K*α radiation, λ = 0.71073 Å
Hall symbol: -P 2ybc	Cell parameters from 9383 reflections
*a* = 8.9063 (6) Å	θ = 2.4–32.4°
*b* = 18.8522 (8) Å	µ = 0.21 mm^−^^1^
*c* = 19.3934 (17) Å	*T* = 120 K
β = 115.314 (6)°	Block, colourless
*V* = 2943.5 (3) Å^3^	0.44 × 0.23 × 0.2 mm
*Z* = 4	

### Data collection

**Table:**

Oxford Diffraction Xcalibur diffractometer with a Sapphire2 (large Be window) detector	4818 independent reflections
graphite	3492 reflections with *I* > 2σ(*I*)
Detector resolution: 8.1883 pixels mm^-1^	*R*_int_ = 0.093
ω scans	θ_max_ = 25.1°, θ_min_ = 2.5°
Absorption correction: multi-scan (*CrysAlis PRO*; Oxford Diffraction, 2009)	*h* = −10→10
*T*_min_ = 0.734, *T*_max_ = 1	*k* = −22→21
14505 measured reflections	*l* = −23→21

### Refinement

**Table:**

Refinement on *F*^2^	Primary atom site location: structure-invariant direct methods
Least-squares matrix: full	Secondary atom site location: difference Fourier map
*R*[*F*^2^ > 2σ(*F*^2^)] = 0.095	Hydrogen site location: inferred from neighbouring sites
*wR*(*F*^2^) = 0.258	H-atom parameters constrained
*S* = 1.13	*w* = 1/[σ^2^(*F*_o_^2^) + (0.0825*P*)^2^ + 14.0784*P*] where *P* = (*F*_o_^2^ + 2*F*_c_^2^)/3
4818 reflections	(Δ/σ)_max_ = 0.001
294 parameters	Δρ_max_ = 0.72 e Å^−^^3^
0 restraints	Δρ_min_ = −0.61 e Å^−^^3^

### Special details

**Table:**

Experimental. Absorption correction: CrysAlisPro, Oxford Diffraction (2009). Empirical absorption correction using spherical harmonics, implemented in SCALE3 ABSPACK scaling algorithm.
Geometry. All s.u.'s (except the s.u. in the dihedral angle between two l.s. planes) are estimated using the full covariance matrix. The cell s.u.'s are taken into account individually in the estimation of s.u.'s in distances, angles and torsion angles; correlations between s.u.'s in cell parameters are only used when they are defined by crystal symmetry. An approximate (isotropic) treatment of cell s.u.'s is used for estimating s.u.'s involving l.s. planes.
Refinement. Refinement of *F*^2^ against ALL reflections. The weighted *R*-factor *wR* and goodness of fit *S* are based on *F*^2^, conventional *R*-factors *R* are based on *F*, with *F* set to zero for negative *F*^2^. The threshold expression of *F*^2^ > 2σ(*F*^2^) is used only for calculating *R*-factors(gt) *etc*. and is not relevant to the choice of reflections for refinement. *R*-factors based on *F*^2^ are statistically about twice as large as those based on *F*, and *R*- factors based on ALL data will be even larger.

### Fractional atomic coordinates and isotropic or equivalent isotropic displacement parameters (Å^2^)

**Table:**

	*x*	*y*	*z*	*U*_iso_*/*U*_eq_	
P1	0.3565 (2)	0.56638 (7)	0.24090 (8)	0.0290 (4)	
P2	0.51867 (18)	0.63068 (7)	0.33654 (8)	0.0269 (4)	
P3	0.33668 (18)	0.64220 (7)	0.15340 (8)	0.0260 (4)	
N1	0.9399 (6)	0.9125 (3)	0.2580 (3)	0.0349 (12)	
Li1	0.5000 (13)	0.7397 (5)	0.2529 (6)	0.032 (2)	
Li2	0.3071 (13)	0.4320 (5)	0.2412 (6)	0.036 (2)	
C1	0.7050 (7)	0.8108 (3)	0.2625 (3)	0.0319 (13)	
C2	0.5670 (8)	0.8291 (3)	0.1954 (3)	0.0369 (14)	
H2	0.5578	0.8235	0.145	0.044*	
C3	0.4453 (8)	0.8569 (3)	0.2148 (4)	0.0450 (17)	
H3	0.3384	0.8727	0.1799	0.054*	
C4	0.5056 (9)	0.8575 (3)	0.2927 (4)	0.0460 (17)	
H4	0.4478	0.8738	0.321	0.055*	
C5	0.6659 (9)	0.8301 (3)	0.3232 (3)	0.0400 (16)	
H5	0.737	0.8252	0.376	0.048*	
C6	0.8712 (9)	0.7859 (4)	0.2700 (5)	0.0536 (19)	
H6A	0.8619	0.7704	0.2206	0.064*	
H6B	0.9075	0.7458	0.3046	0.064*	
C7	0.9994 (9)	0.8457 (4)	0.3003 (5)	0.0528 (19)	
H7A	1.0261	0.8538	0.3537	0.063*	
H7B	1.1005	0.8312	0.2969	0.063*	
C8	1.0617 (9)	0.9682 (4)	0.2961 (4)	0.0498 (17)	
H8A	1.0249	1.0119	0.2687	0.075*	
H8B	1.167	0.9551	0.2973	0.075*	
H8C	1.0731	0.974	0.3473	0.075*	
C9	0.9215 (10)	0.9045 (4)	0.1805 (4)	0.0552 (19)	
H9A	1.0235	0.8871	0.1812	0.083*	
H9B	0.8952	0.9496	0.1551	0.083*	
H9C	0.8336	0.8715	0.1537	0.083*	
C10	0.7138 (8)	0.5767 (4)	0.3918 (4)	0.0415 (15)	
C11	0.6889 (11)	0.5100 (4)	0.4292 (4)	0.060 (2)	
H11A	0.6598	0.5226	0.4699	0.09*	
H11B	0.7898	0.4828	0.4493	0.09*	
H11C	0.6012	0.4822	0.3921	0.09*	
C12	0.8439 (10)	0.6256 (5)	0.4510 (4)	0.062 (2)	
H12A	0.859	0.667	0.426	0.093*	
H12B	0.9475	0.6007	0.4753	0.093*	
H12C	0.8064	0.6394	0.4887	0.093*	
C13	0.7840 (9)	0.5558 (4)	0.3354 (4)	0.0536 (19)	
H13A	0.8041	0.5977	0.3125	0.08*	
H13B	0.7057	0.526	0.2964	0.08*	
H13C	0.8864	0.5305	0.3617	0.08*	
C14	0.4030 (9)	0.6415 (4)	0.3992 (3)	0.0427 (16)	
C15	0.3039 (12)	0.5759 (5)	0.4005 (5)	0.067 (2)	
H15A	0.2249	0.5648	0.3495	0.1*	
H15B	0.2463	0.5851	0.4316	0.1*	
H15C	0.3781	0.5366	0.4213	0.1*	
C16	0.5123 (11)	0.6646 (5)	0.4799 (4)	0.063 (2)	
H16A	0.5816	0.7032	0.4789	0.094*	
H16B	0.5807	0.6255	0.5076	0.094*	
H16C	0.4441	0.6796	0.5043	0.094*	
C17	0.2809 (10)	0.7008 (4)	0.3627 (4)	0.055 (2)	
H17A	0.2075	0.6878	0.3113	0.082*	
H17B	0.34	0.7432	0.3621	0.082*	
H17C	0.2174	0.709	0.3914	0.082*	
C18	0.4247 (8)	0.5971 (3)	0.0903 (3)	0.0340 (13)	
C19	0.3811 (13)	0.5198 (4)	0.0759 (5)	0.062 (2)	
H19A	0.265	0.5151	0.0429	0.093*	
H19B	0.4051	0.4964	0.1235	0.093*	
H19C	0.4453	0.4985	0.0523	0.093*	
C20	0.3761 (12)	0.6371 (4)	0.0141 (4)	0.059 (2)	
H20A	0.4116	0.6856	0.0243	0.089*	
H20B	0.2578	0.6355	−0.015	0.089*	
H20C	0.4289	0.615	−0.0144	0.089*	
C21	0.6111 (10)	0.6071 (5)	0.1337 (4)	0.064 (2)	
H21A	0.6354	0.6565	0.1452	0.096*	
H21B	0.6649	0.5907	0.103	0.096*	
H21C	0.651	0.5804	0.1803	0.096*	
C22	0.1056 (7)	0.6575 (3)	0.0937 (3)	0.0343 (14)	
C23	0.0350 (9)	0.6805 (4)	0.1482 (4)	0.0488 (17)	
H23A	0.0928	0.722	0.1753	0.073*	
H23B	0.0481	0.643	0.1838	0.073*	
H23C	−0.0808	0.6912	0.1201	0.073*	
C24	0.0087 (9)	0.5933 (4)	0.0490 (4)	0.055 (2)	
H24A	0.0451	0.5812	0.0105	0.082*	
H24B	−0.1077	0.6043	0.0252	0.082*	
H24C	0.0275	0.5539	0.083	0.082*	
C25	0.0831 (10)	0.7205 (4)	0.0392 (4)	0.0540 (19)	
H25A	0.1163	0.7065	0.0002	0.081*	
H25B	0.1505	0.7596	0.0674	0.081*	
H25C	−0.0314	0.7346	0.0161	0.081*	

### Atomic displacement parameters (Å^2^)

**Table:**

	*U*^11^	*U*^22^	*U*^33^	*U*^12^	*U*^13^	*U*^23^
P1	0.0402 (9)	0.0167 (7)	0.0282 (8)	−0.0029 (6)	0.0129 (7)	0.0010 (5)
P2	0.0360 (8)	0.0175 (7)	0.0253 (8)	0.0028 (6)	0.0113 (6)	0.0008 (5)
P3	0.0339 (8)	0.0165 (7)	0.0256 (8)	0.0011 (6)	0.0108 (6)	0.0011 (5)
N1	0.035 (3)	0.026 (3)	0.047 (3)	−0.002 (2)	0.021 (2)	−0.003 (2)
Li1	0.041 (5)	0.018 (5)	0.040 (6)	−0.001 (4)	0.021 (4)	−0.001 (4)
Li2	0.043 (6)	0.019 (5)	0.049 (6)	−0.004 (4)	0.023 (5)	0.002 (4)
C1	0.036 (3)	0.012 (3)	0.048 (4)	0.000 (2)	0.017 (3)	0.001 (2)
C2	0.051 (4)	0.027 (3)	0.034 (3)	−0.015 (3)	0.018 (3)	−0.001 (2)
C3	0.035 (3)	0.020 (3)	0.072 (5)	−0.005 (3)	0.015 (3)	0.012 (3)
C4	0.059 (4)	0.020 (3)	0.074 (5)	−0.008 (3)	0.043 (4)	−0.013 (3)
C5	0.060 (4)	0.025 (3)	0.030 (3)	−0.018 (3)	0.015 (3)	−0.004 (2)
C6	0.046 (4)	0.027 (4)	0.094 (6)	0.005 (3)	0.035 (4)	0.004 (3)
C7	0.035 (4)	0.044 (4)	0.076 (5)	−0.001 (3)	0.022 (4)	0.010 (4)
C8	0.044 (4)	0.040 (4)	0.061 (5)	−0.007 (3)	0.018 (3)	−0.008 (3)
C9	0.062 (5)	0.054 (5)	0.059 (5)	−0.011 (4)	0.035 (4)	−0.008 (3)
C10	0.043 (4)	0.041 (4)	0.031 (3)	0.006 (3)	0.007 (3)	−0.003 (3)
C11	0.087 (6)	0.038 (4)	0.042 (4)	0.027 (4)	0.016 (4)	0.017 (3)
C12	0.049 (4)	0.061 (5)	0.049 (5)	0.001 (4)	−0.003 (4)	−0.012 (4)
C13	0.044 (4)	0.059 (5)	0.053 (5)	0.016 (3)	0.016 (3)	−0.003 (3)
C14	0.051 (4)	0.053 (4)	0.027 (3)	0.012 (3)	0.018 (3)	0.005 (3)
C15	0.089 (6)	0.074 (6)	0.061 (5)	0.005 (5)	0.054 (5)	0.011 (4)
C16	0.081 (6)	0.069 (6)	0.034 (4)	0.026 (4)	0.019 (4)	−0.006 (3)
C17	0.064 (5)	0.062 (5)	0.045 (4)	0.026 (4)	0.029 (4)	0.002 (3)
C18	0.048 (4)	0.024 (3)	0.032 (3)	0.006 (3)	0.019 (3)	0.000 (2)
C19	0.108 (7)	0.033 (4)	0.068 (5)	0.007 (4)	0.058 (5)	−0.008 (3)
C20	0.091 (6)	0.053 (5)	0.045 (4)	0.004 (4)	0.040 (4)	0.009 (3)
C21	0.057 (5)	0.088 (7)	0.054 (5)	0.013 (4)	0.031 (4)	−0.003 (4)
C22	0.033 (3)	0.031 (3)	0.030 (3)	0.005 (2)	0.005 (3)	0.001 (2)
C23	0.038 (4)	0.052 (4)	0.048 (4)	0.011 (3)	0.010 (3)	−0.002 (3)
C24	0.048 (4)	0.048 (5)	0.048 (4)	−0.008 (3)	0.001 (3)	−0.006 (3)
C25	0.058 (4)	0.044 (4)	0.048 (4)	0.015 (3)	0.010 (3)	0.017 (3)

### Geometric parameters (Å, °)

**Table:**

P1—P3	2.168 (2)	C9—H9C	0.96
P1—P2	2.169 (2)	C10—C11	1.515 (10)
P1—Li2	2.571 (10)	C10—C13	1.527 (10)
P2—C10	1.900 (7)	C10—C12	1.540 (10)
P2—C14	1.910 (7)	C11—H11A	0.96
P2—Li1	2.579 (10)	C11—H11B	0.96
P3—C22	1.903 (6)	C11—H11C	0.96
P3—C18	1.912 (6)	C12—H12A	0.96
P3—Li1	2.611 (10)	C12—H12B	0.96
N1—C9	1.448 (8)	C12—H12C	0.96
N1—C8	1.463 (8)	C13—H13A	0.96
N1—C7	1.472 (9)	C13—H13B	0.96
N1—Li2^i^	2.237 (12)	C13—H13C	0.96
Li1—C1	2.208 (11)	C14—C17	1.508 (10)
Li1—C2	2.238 (11)	C14—C16	1.511 (10)
Li1—C5	2.286 (11)	C14—C15	1.525 (12)
Li1—C3	2.314 (11)	C15—H15A	0.96
Li1—C4	2.345 (11)	C15—H15B	0.96
Li2—N1^ii^	2.237 (12)	C15—H15C	0.96
Li2—C1^ii^	2.287 (11)	C16—H16A	0.96
Li2—C2^ii^	2.314 (12)	C16—H16B	0.96
Li2—C5^ii^	2.359 (12)	C16—H16C	0.96
Li2—C3^ii^	2.447 (12)	C17—H17A	0.96
Li2—C4^ii^	2.475 (12)	C17—H17B	0.96
C1—C2	1.398 (9)	C17—H17C	0.96
C1—C5	1.410 (9)	C18—C19	1.503 (10)
C1—C6	1.500 (9)	C18—C21	1.518 (11)
C1—Li2^i^	2.287 (11)	C18—C20	1.547 (9)
C2—C3	1.394 (10)	C19—H19A	0.96
C2—Li2^i^	2.314 (12)	C19—H19B	0.96
C2—H2	0.95	C19—H19C	0.96
C3—C4	1.370 (10)	C20—H20A	0.96
C3—Li2^i^	2.447 (12)	C20—H20B	0.96
C3—H3	0.95	C20—H20C	0.96
C4—C5	1.390 (10)	C21—H21A	0.96
C4—Li2^i^	2.475 (12)	C21—H21B	0.96
C4—H4	0.95	C21—H21C	0.96
C5—Li2^i^	2.359 (12)	C22—C23	1.506 (9)
C5—H5	0.9501	C22—C24	1.524 (9)
C6—C7	1.533 (10)	C22—C25	1.546 (9)
C6—H6A	0.97	C23—H23A	0.96
C6—H6B	0.97	C23—H23B	0.96
C7—H7A	0.97	C23—H23C	0.96
C7—H7B	0.97	C24—H24A	0.96
C8—H8A	0.96	C24—H24B	0.96
C8—H8B	0.96	C24—H24C	0.96
C8—H8C	0.96	C25—H25A	0.96
C9—H9A	0.96	C25—H25B	0.96
C9—H9B	0.96	C25—H25C	0.96
			
P3—P1—P2	95.87 (8)	C7—C6—H6B	109.6
P3—P1—Li2	134.0 (2)	H6A—C6—H6B	108.1
P2—P1—Li2	126.9 (2)	N1—C7—C6	112.6 (6)
C10—P2—C14	108.1 (3)	N1—C7—H7A	109.1
C10—P2—P1	107.3 (2)	C6—C7—H7A	109.1
C14—P2—P1	105.1 (2)	N1—C7—H7B	109.1
C10—P2—Li1	125.4 (3)	C6—C7—H7B	109.1
C14—P2—Li1	114.1 (3)	H7A—C7—H7B	107.8
P1—P2—Li1	93.8 (2)	N1—C8—H8A	109.5
C22—P3—C18	108.1 (3)	N1—C8—H8B	109.5
C22—P3—P1	106.3 (2)	H8A—C8—H8B	109.5
C18—P3—P1	106.83 (19)	N1—C8—H8C	109.5
C22—P3—Li1	117.3 (3)	H8A—C8—H8C	109.5
C18—P3—Li1	122.3 (3)	H8B—C8—H8C	109.5
P1—P3—Li1	93.0 (2)	N1—C9—H9A	109.5
C9—N1—C8	109.2 (5)	N1—C9—H9B	109.5
C9—N1—C7	110.1 (6)	H9A—C9—H9B	109.5
C8—N1—C7	108.6 (5)	N1—C9—H9C	109.5
C9—N1—Li2^i^	110.5 (5)	H9A—C9—H9C	109.5
C8—N1—Li2^i^	113.4 (5)	H9B—C9—H9C	109.5
C7—N1—Li2^i^	104.9 (5)	C11—C10—C13	108.5 (6)
C1—Li1—C2	36.6 (3)	C11—C10—C12	110.9 (6)
C1—Li1—C5	36.5 (3)	C13—C10—C12	106.3 (6)
C2—Li1—C5	59.6 (3)	C11—C10—P2	115.4 (5)
C1—Li1—C3	60.0 (3)	C13—C10—P2	106.9 (4)
C2—Li1—C3	35.6 (3)	C12—C10—P2	108.4 (5)
C5—Li1—C3	58.2 (3)	C10—C11—H11A	109.5
C1—Li1—C4	59.8 (3)	C10—C11—H11B	109.5
C2—Li1—C4	58.6 (3)	H11A—C11—H11B	109.5
C5—Li1—C4	34.9 (3)	C10—C11—H11C	109.5
C3—Li1—C4	34.2 (3)	H11A—C11—H11C	109.5
C1—Li1—P2	127.0 (5)	H11B—C11—H11C	109.5
C2—Li1—P2	162.6 (5)	C10—C12—H12A	109.5
C5—Li1—P2	111.6 (4)	C10—C12—H12B	109.5
C3—Li1—P2	155.4 (5)	H12A—C12—H12B	109.5
C4—Li1—P2	124.1 (4)	C10—C12—H12C	109.5
C1—Li1—P3	132.0 (5)	H12A—C12—H12C	109.5
C2—Li1—P3	110.2 (4)	H12B—C12—H12C	109.5
C5—Li1—P3	168.4 (5)	C10—C13—H13A	109.5
C3—Li1—P3	117.5 (4)	C10—C13—H13B	109.5
C4—Li1—P3	146.8 (5)	H13A—C13—H13B	109.5
P2—Li1—P3	76.7 (3)	C10—C13—H13C	109.5
N1^ii^—Li2—C1^ii^	78.6 (4)	H13A—C13—H13C	109.5
N1^ii^—Li2—C2^ii^	96.4 (4)	H13B—C13—H13C	109.5
C1^ii^—Li2—C2^ii^	35.4 (3)	C17—C14—C16	106.8 (6)
N1^ii^—Li2—C5^ii^	101.1 (4)	C17—C14—C15	107.5 (7)
C1^ii^—Li2—C5^ii^	35.3 (3)	C16—C14—C15	109.7 (6)
C2^ii^—Li2—C5^ii^	57.5 (3)	C17—C14—P2	104.9 (4)
N1^ii^—Li2—C3^ii^	130.1 (5)	C16—C14—P2	113.9 (5)
C1^ii^—Li2—C3^ii^	57.0 (3)	C15—C14—P2	113.5 (5)
C2^ii^—Li2—C3^ii^	33.9 (3)	C14—C15—H15A	109.5
C5^ii^—Li2—C3^ii^	55.4 (3)	C14—C15—H15B	109.5
N1^ii^—Li2—C4^ii^	133.2 (5)	H15A—C15—H15B	109.5
C1^ii^—Li2—C4^ii^	56.8 (3)	C14—C15—H15C	109.5
C2^ii^—Li2—C4^ii^	55.7 (3)	H15A—C15—H15C	109.5
C5^ii^—Li2—C4^ii^	33.3 (3)	H15B—C15—H15C	109.5
C3^ii^—Li2—C4^ii^	32.3 (3)	C14—C16—H16A	109.5
N1^ii^—Li2—P1	109.4 (4)	C14—C16—H16B	109.5
C1^ii^—Li2—P1	171.8 (5)	H16A—C16—H16B	109.5
C2^ii^—Li2—P1	141.5 (5)	C14—C16—H16C	109.5
C5^ii^—Li2—P1	138.1 (5)	H16A—C16—H16C	109.5
C3^ii^—Li2—P1	116.2 (4)	H16B—C16—H16C	109.5
C4^ii^—Li2—P1	115.0 (4)	C14—C17—H17A	109.5
C2—C1—C5	106.4 (6)	C14—C17—H17B	109.5
C2—C1—C6	127.2 (6)	H17A—C17—H17B	109.5
C5—C1—C6	125.9 (6)	C14—C17—H17C	109.5
C2—C1—Li1	72.9 (4)	H17A—C17—H17C	109.5
C5—C1—Li1	74.7 (4)	H17B—C17—H17C	109.5
C6—C1—Li1	124.3 (5)	C19—C18—C21	111.0 (7)
C2—C1—Li2^i^	73.4 (4)	C19—C18—C20	110.5 (6)
C5—C1—Li2^i^	75.2 (4)	C21—C18—C20	105.2 (6)
C6—C1—Li2^i^	110.3 (5)	C19—C18—P3	113.9 (5)
Li1—C1—Li2^i^	125.3 (5)	C21—C18—P3	103.9 (4)
C3—C2—C1	108.4 (6)	C20—C18—P3	111.8 (4)
C3—C2—Li1	75.2 (4)	C18—C19—H19A	109.5
C1—C2—Li1	70.5 (4)	C18—C19—H19B	109.5
C3—C2—Li2^i^	78.3 (5)	H19A—C19—H19B	109.5
C1—C2—Li2^i^	71.2 (4)	C18—C19—H19C	109.5
Li1—C2—Li2^i^	122.6 (4)	H19A—C19—H19C	109.5
C3—C2—H2	125.8	H19B—C19—H19C	109.5
C1—C2—H2	125.8	C18—C20—H20A	109.5
Li1—C2—H2	120.3	C18—C20—H20B	109.5
Li2^i^—C2—H2	116.6	H20A—C20—H20B	109.5
C4—C3—C2	108.5 (6)	C18—C20—H20C	109.5
C4—C3—Li1	74.1 (4)	H20A—C20—H20C	109.5
C2—C3—Li1	69.2 (4)	H20B—C20—H20C	109.5
C4—C3—Li2^i^	75.0 (5)	C18—C21—H21A	109.5
C2—C3—Li2^i^	67.8 (4)	C18—C21—H21B	109.5
Li1—C3—Li2^i^	113.9 (4)	H21A—C21—H21B	109.5
C4—C3—H3	125.7	C18—C21—H21C	109.5
C2—C3—H3	125.7	H21A—C21—H21C	109.5
Li1—C3—H3	122.5	H21B—C21—H21C	109.5
Li2^i^—C3—H3	123.1	C23—C22—C24	108.9 (6)
C3—C4—C5	108.2 (6)	C23—C22—C25	106.7 (6)
C3—C4—Li1	71.7 (4)	C24—C22—C25	110.2 (5)
C5—C4—Li1	70.2 (4)	C23—C22—P3	106.5 (4)
C3—C4—Li2^i^	72.7 (4)	C24—C22—P3	115.4 (5)
C5—C4—Li2^i^	68.8 (4)	C25—C22—P3	108.8 (5)
Li1—C4—Li2^i^	111.8 (4)	C22—C23—H23A	109.5
C3—C4—H4	125.9	C22—C23—H23B	109.5
C5—C4—H4	125.9	H23A—C23—H23B	109.5
Li1—C4—H4	123.8	C22—C23—H23C	109.5
Li2^i^—C4—H4	124.2	H23A—C23—H23C	109.5
C4—C5—C1	108.4 (6)	H23B—C23—H23C	109.5
C4—C5—Li1	74.9 (4)	C22—C24—H24A	109.5
C1—C5—Li1	68.7 (4)	C22—C24—H24B	109.5
C4—C5—Li2^i^	77.9 (5)	H24A—C24—H24B	109.5
C1—C5—Li2^i^	69.5 (4)	C22—C24—H24C	109.5
Li1—C5—Li2^i^	118.5 (4)	H24A—C24—H24C	109.5
C4—C5—H5	125.8	H24B—C24—H24C	109.5
C1—C5—H5	125.8	C22—C25—H25A	109.5
Li1—C5—H5	122.3	C22—C25—H25B	109.5
Li2^i^—C5—H5	118.5	H25A—C25—H25B	109.5
C1—C6—C7	110.4 (6)	C22—C25—H25C	109.5
C1—C6—H6A	109.6	H25A—C25—H25C	109.5
C7—C6—H6A	109.6	H25B—C25—H25C	109.5
C1—C6—H6B	109.6		
			
P3—P1—P2—C10	−122.7 (2)	C5—Li1—C3—C4	−36.5 (4)
Li2—P1—P2—C10	39.2 (4)	P2—Li1—C3—C4	35.0 (12)
P3—P1—P2—C14	122.5 (2)	P3—Li1—C3—C4	155.9 (6)
Li2—P1—P2—C14	−75.6 (4)	C1—Li1—C3—C2	38.2 (4)
P3—P1—P2—Li1	6.3 (2)	C5—Li1—C3—C2	81.0 (4)
Li2—P1—P2—Li1	168.2 (4)	C4—Li1—C3—C2	117.5 (6)
P2—P1—P3—C22	−125.8 (2)	P2—Li1—C3—C2	152.5 (12)
Li2—P1—P3—C22	74.4 (4)	P3—Li1—C3—C2	−86.6 (5)
P2—P1—P3—C18	118.9 (2)	C1—Li1—C3—Li2^i^	−14.2 (5)
Li2—P1—P3—C18	−40.9 (4)	C2—Li1—C3—Li2^i^	−52.4 (5)
P2—P1—P3—Li1	−6.2 (2)	C5—Li1—C3—Li2^i^	28.6 (5)
Li2—P1—P3—Li1	−166.0 (4)	C4—Li1—C3—Li2^i^	65.1 (5)
C10—P2—Li1—C1	−24.0 (7)	P2—Li1—C3—Li2^i^	100.1 (12)
C14—P2—Li1—C1	113.2 (5)	P3—Li1—C3—Li2^i^	−139.0 (5)
P1—P2—Li1—C1	−138.4 (5)	C2—C3—C4—C5	0.0 (7)
C10—P2—Li1—C2	−6.4 (17)	Li1—C3—C4—C5	61.0 (5)
C14—P2—Li1—C2	130.8 (15)	Li2^i^—C3—C4—C5	−59.9 (5)
P1—P2—Li1—C2	−120.8 (16)	C2—C3—C4—Li1	−61.0 (5)
C10—P2—Li1—C5	−62.4 (5)	Li2^i^—C3—C4—Li1	−120.9 (4)
C14—P2—Li1—C5	74.8 (5)	C2—C3—C4—Li2^i^	59.8 (5)
P1—P2—Li1—C5	−176.7 (4)	Li1—C3—C4—Li2^i^	120.9 (4)
C10—P2—Li1—C3	−122.4 (11)	C1—Li1—C4—C3	80.2 (4)
C14—P2—Li1—C3	14.8 (13)	C2—Li1—C4—C3	37.3 (4)
P1—P2—Li1—C3	123.2 (12)	C5—Li1—C4—C3	118.0 (6)
C10—P2—Li1—C4	−99.5 (6)	P2—Li1—C4—C3	−163.3 (6)
C14—P2—Li1—C4	37.7 (6)	P3—Li1—C4—C3	−41.4 (9)
P1—P2—Li1—C4	146.1 (5)	C1—Li1—C4—C5	−37.8 (4)
C10—P2—Li1—P3	109.1 (3)	C2—Li1—C4—C5	−80.7 (5)
C14—P2—Li1—P3	−113.7 (3)	C3—Li1—C4—C5	−118.0 (6)
P1—P2—Li1—P3	−5.3 (2)	P2—Li1—C4—C5	78.7 (6)
C22—P3—Li1—C1	−116.3 (6)	P3—Li1—C4—C5	−159.4 (9)
C18—P3—Li1—C1	21.5 (7)	C1—Li1—C4—Li2^i^	18.2 (4)
P1—P3—Li1—C1	133.6 (6)	C2—Li1—C4—Li2^i^	−24.7 (4)
C22—P3—Li1—C2	−81.3 (5)	C5—Li1—C4—Li2^i^	56.0 (5)
C18—P3—Li1—C2	56.5 (5)	C3—Li1—C4—Li2^i^	−62.0 (5)
P1—P3—Li1—C2	168.6 (4)	P2—Li1—C4—Li2^i^	134.8 (5)
C22—P3—Li1—C5	−108 (2)	P3—Li1—C4—Li2^i^	−103.4 (9)
C18—P3—Li1—C5	30 (3)	C3—C4—C5—C1	−1.0 (7)
P1—P3—Li1—C5	142 (2)	Li1—C4—C5—C1	60.9 (5)
C22—P3—Li1—C3	−43.1 (6)	Li2^i^—C4—C5—C1	−63.4 (5)
C18—P3—Li1—C3	94.7 (5)	C3—C4—C5—Li1	−61.9 (5)
P1—P3—Li1—C3	−153.2 (4)	Li2^i^—C4—C5—Li1	−124.3 (4)
C22—P3—Li1—C4	−18.3 (10)	C3—C4—C5—Li2^i^	62.4 (5)
C18—P3—Li1—C4	119.5 (8)	Li1—C4—C5—Li2^i^	124.3 (4)
P1—P3—Li1—C4	−128.4 (8)	C2—C1—C5—C4	1.6 (7)
C22—P3—Li1—P2	115.4 (3)	C6—C1—C5—C4	173.5 (6)
C18—P3—Li1—P2	−106.8 (3)	Li1—C1—C5—C4	−64.9 (5)
P1—P3—Li1—P2	5.3 (2)	Li2^i^—C1—C5—C4	68.9 (5)
P3—P1—Li2—N1^ii^	−101.3 (4)	C2—C1—C5—Li1	66.5 (5)
P2—P1—Li2—N1^ii^	104.1 (4)	C6—C1—C5—Li1	−121.6 (6)
P3—P1—Li2—C2^ii^	129.4 (7)	Li2^i^—C1—C5—Li1	133.8 (4)
P2—P1—Li2—C2^ii^	−25.2 (9)	C2—C1—C5—Li2^i^	−67.3 (5)
P3—P1—Li2—C5^ii^	33.1 (9)	C6—C1—C5—Li2^i^	104.6 (7)
P2—P1—Li2—C5^ii^	−121.5 (6)	Li1—C1—C5—Li2^i^	−133.8 (4)
P3—P1—Li2—C3^ii^	99.6 (5)	C1—Li1—C5—C4	117.2 (6)
P2—P1—Li2—C3^ii^	−55.0 (6)	C2—Li1—C5—C4	77.6 (5)
P3—P1—Li2—C4^ii^	63.7 (6)	C3—Li1—C5—C4	35.7 (4)
P2—P1—Li2—C4^ii^	−91.0 (5)	P2—Li1—C5—C4	−119.2 (5)
C5—Li1—C1—C2	−112.9 (6)	P3—Li1—C5—C4	107 (2)
C3—Li1—C1—C2	−37.1 (4)	C2—Li1—C5—C1	−39.6 (4)
C4—Li1—C1—C2	−76.8 (4)	C3—Li1—C5—C1	−81.4 (4)
P2—Li1—C1—C2	171.2 (6)	C4—Li1—C5—C1	−117.2 (6)
P3—Li1—C1—C2	64.3 (6)	P2—Li1—C5—C1	123.7 (5)
C2—Li1—C1—C5	112.9 (6)	P3—Li1—C5—C1	−10 (2)
C3—Li1—C1—C5	75.8 (4)	C1—Li1—C5—Li2^i^	50.3 (5)
C4—Li1—C1—C5	36.1 (4)	C2—Li1—C5—Li2^i^	10.7 (5)
P2—Li1—C1—C5	−75.8 (6)	C3—Li1—C5—Li2^i^	−31.1 (5)
P3—Li1—C1—C5	177.2 (7)	C4—Li1—C5—Li2^i^	−66.8 (5)
C2—Li1—C1—C6	−123.8 (8)	P2—Li1—C5—Li2^i^	174.0 (4)
C5—Li1—C1—C6	123.3 (7)	P3—Li1—C5—Li2^i^	40 (3)
C3—Li1—C1—C6	−160.9 (7)	C2—C1—C6—C7	103.9 (8)
C4—Li1—C1—C6	159.4 (7)	C5—C1—C6—C7	−66.4 (9)
P2—Li1—C1—C6	47.5 (9)	Li1—C1—C6—C7	−162.1 (6)
P3—Li1—C1—C6	−59.5 (9)	Li2^i^—C1—C6—C7	19.7 (8)
C2—Li1—C1—Li2^i^	54.2 (5)	C9—N1—C7—C6	−66.8 (8)
C5—Li1—C1—Li2^i^	−58.8 (6)	C8—N1—C7—C6	173.6 (6)
C3—Li1—C1—Li2^i^	17.1 (5)	Li2^i^—N1—C7—C6	52.1 (8)
C4—Li1—C1—Li2^i^	−22.7 (5)	C1—C6—C7—N1	−49.6 (9)
P2—Li1—C1—Li2^i^	−134.6 (5)	C14—P2—C10—C11	47.7 (6)
P3—Li1—C1—Li2^i^	118.5 (6)	P1—P2—C10—C11	−65.1 (5)
C5—C1—C2—C3	−1.6 (6)	Li1—P2—C10—C11	−173.0 (5)
C6—C1—C2—C3	−173.4 (6)	C14—P2—C10—C13	168.4 (5)
Li1—C1—C2—C3	66.2 (5)	P1—P2—C10—C13	55.6 (5)
Li2^i^—C1—C2—C3	−70.2 (5)	Li1—P2—C10—C13	−52.3 (6)
C5—C1—C2—Li1	−67.8 (5)	C14—P2—C10—C12	−77.4 (6)
C6—C1—C2—Li1	120.4 (7)	P1—P2—C10—C12	169.8 (5)
Li2^i^—C1—C2—Li1	−136.4 (4)	Li1—P2—C10—C12	61.9 (6)
C5—C1—C2—Li2^i^	68.5 (5)	C10—P2—C14—C17	164.2 (5)
C6—C1—C2—Li2^i^	−103.2 (7)	P1—P2—C14—C17	−81.5 (5)
Li1—C1—C2—Li2^i^	136.4 (4)	Li1—P2—C14—C17	19.8 (6)
C1—Li1—C2—C3	−116.1 (6)	C10—P2—C14—C16	47.7 (6)
C5—Li1—C2—C3	−76.7 (5)	P1—P2—C14—C16	162.0 (5)
C4—Li1—C2—C3	−35.8 (4)	Li1—P2—C14—C16	−96.6 (6)
P2—Li1—C2—C3	−140.1 (16)	C10—P2—C14—C15	−78.7 (6)
P3—Li1—C2—C3	109.4 (5)	P1—P2—C14—C15	35.6 (6)
C5—Li1—C2—C1	39.5 (4)	Li1—P2—C14—C15	136.9 (5)
C3—Li1—C2—C1	116.1 (6)	C22—P3—C18—C19	−75.4 (6)
C4—Li1—C2—C1	80.4 (4)	P1—P3—C18—C19	38.6 (6)
P2—Li1—C2—C1	−23.9 (16)	Li1—P3—C18—C19	143.5 (5)
P3—Li1—C2—C1	−134.5 (5)	C22—P3—C18—C21	163.7 (5)
C1—Li1—C2—Li2^i^	−50.9 (5)	P1—P3—C18—C21	−82.2 (5)
C5—Li1—C2—Li2^i^	−11.4 (5)	Li1—P3—C18—C21	22.6 (6)
C3—Li1—C2—Li2^i^	65.3 (6)	C22—P3—C18—C20	50.8 (6)
C4—Li1—C2—Li2^i^	29.5 (5)	P1—P3—C18—C20	164.8 (5)
P2—Li1—C2—Li2^i^	−74.8 (17)	Li1—P3—C18—C20	−90.3 (6)
P3—Li1—C2—Li2^i^	174.6 (4)	C18—P3—C22—C23	170.0 (4)
C1—C2—C3—C4	1.0 (7)	P1—P3—C22—C23	55.6 (5)
Li1—C2—C3—C4	64.2 (5)	Li1—P3—C22—C23	−46.7 (6)
Li2^i^—C2—C3—C4	−64.4 (5)	C18—P3—C22—C24	49.0 (6)
C1—C2—C3—Li1	−63.1 (5)	P1—P3—C22—C24	−65.4 (5)
Li2^i^—C2—C3—Li1	−128.6 (4)	Li1—P3—C22—C24	−167.6 (5)
C1—C2—C3—Li2^i^	65.5 (5)	C18—P3—C22—C25	−75.4 (5)
Li1—C2—C3—Li2^i^	128.6 (4)	P1—P3—C22—C25	170.2 (4)
C1—Li1—C3—C4	−79.3 (5)	Li1—P3—C22—C25	68.0 (5)
C2—Li1—C3—C4	−117.5 (6)		

Symmetry codes: (i) −*x*+1, *y*+1/2, −*z*+1/2; (ii) −*x*+1, *y*−1/2, −*z*+1/2.

## References

[bb1] Chojnacki, J., Grubba, R., Kugiel-Rachwalska, B. & Pikies, J. (2007). *Polyhedron*, **26**, 1579–1582.

[bb2] Farrugia, L. J. (1997). *J. Appl. Cryst.* **30**, 565.

[bb3] Farrugia, L. J. (1999). *J. Appl. Cryst.* **32**, 837–838.

[bb4] Kovacs, I., Krautscheid, H., Matern, E., Sattler, E., Fritz, G., Hönle, W., Borrmann, H. & von Schnering, H. G. (1996*a*). *Z. Anorg. Allg. Chem.* **622**, 1564–1572.

[bb5] Kovacs, I., Matern, E. & Fritz, G. Z. (1996*b*). *Z. Anorg. Allg. Chem.* **622**, 935–941.

[bb6] Kunz, K., Pflug, J., Bertuleit, A., Frohlich, R., Wegelius, E., Erker, G. & Wurthwein, E.-U. (2000). *Organometallics*, **19**, 4208–4216.

[bb7] Oxford Diffraction (2009). *CrysAlis PRO* Oxford Diffraction Ltd, Yarnton, England.

[bb8] Sheldrick, G. M. (2008). *Acta Cryst.* A**64**, 112–122. 10.1107/S0108767307043930 18156677

